# Euglycemic Diabetic Ketoacidosis in the Setting of SGLT2 Inhibitor Use and Hypertriglyceridemia: A Case Report and Review of Literature

**DOI:** 10.7759/cureus.4384

**Published:** 2019-04-04

**Authors:** Kushani Gajjar, Pooja Luthra

**Affiliations:** 1 Internal Medicine, University of Connecticut Health Center, Farmington, USA

**Keywords:** euglycemic dka, sglt2 inhibitor, dapagliflozin, euglycemic diabetic ketoacidosis

## Abstract

We describe the case report of a patient with euglycemic diabetic ketoacidosis (euDKA), in the setting of sodium-glucose cotransporter-2 (SGLT2) inhibitor use, complicated by hypertriglyceridemia (HTG).

A 28-year-old female with a history of gestational diabetes mellitus and subsequent type 2 diabetes mellitus (T2DM) on dapagliflozin and metformin presented with a one-week history of polyuria, poor appetite, and vomiting. On admission, serum glucose was 111 mg/dl, bicarbonate 18 mmol/l, anion gap 20, triglycerides 508 mg/dL, and venous pH 7.27. Serum ketone levels could not be assessed, as blood samples kept hemolyzing due to significant lipemia. The patient was initially admitted for starvation ketosis. However, serum chemistry obtained six hours after presentation revealed no change in the anion gap and a rise in triglycerides. She was treated with an insulin drip for euDKA and HTG with the resolution of the clinical picture.

We performed a literature review of this topic and discuss the pathophysiology, diagnosis, management, and prevention of SGLT2-inhibitor-induced euDKA.

## Introduction

Diabetic ketoacidosis (DKA) is a medical emergency characterized by the triad of hyperglycemia (blood sugar >250 mg/dl), metabolic acidosis (arterial pH <7.3 and serum bicarbonate <18 mEq/L), and ketosis. Rarely, patients can present with blood glucose (BG) levels of less than 200 mg/dl, which is defined as euglycemic DKA (euDKA). There is an established, though rare, association of DKA with normal glucose values or euDKA with the use of sodium-glucose cotransporter-2 inhibitors (SGLT2 inhibitors) [1-7].

We describe the case report of a patient with euDKA complicated by hypertriglyceridemia (HTG) in the setting of SGLT2 inhibitor use. To our knowledge, this is the first case report describing euDKA in the presence of hypertriglyceridemia.

## Case presentation

A 28-year-old female with a history of gestational diabetes mellitus diagnosed eight years prior to presentation and subsequent type two diabetes mellitus (T2DM), one prior episode of HTG-induced pancreatitis three years prior to presentation, and obesity with a body mass index (BMI) of 33.5 kg/m^2^, presented with a one-week history of polyuria, polydipsia, poor appetite, and vomiting. Two weeks prior to presentation, she was treated with a five-day course of amoxicillin for a respiratory tract infection. She was on metformin, glipizide, and dapagliflozin for T2DM and atorvastatin and gemfibrozil for HTG. She had been on dapagliflozin for six months at the time of presentation. Physical examination on presentation was significant for dry oral mucosa; significantly, her abdominal examination was benign with no tenderness, guarding, or rigidity. Pertinent laboratory findings on admission were: serum glucose 111 mg/dl, bicarbonate 18 mmol/l, anion gap 20, creatinine 0.4 mg/dL, triglycerides 508 mg/dL, total cholesterol 122 mg/dL, glycated hemoglobin (HbA1c) 10%, and venous pH 7.27. Serum lipase was normal at 43 U/L. Serum acetone levels could not be assessed as blood samples kept hemolyzing due to significant lipemia. The patient was initially admitted for starvation ketosis, as she reported poor oral intake for three days prior to admission. However, serum chemistry obtained six hours after presentation revealed her glucose was 186 mg/dL, the anion gap was still elevated at 21, serum bicarbonate was 16 mmol/L, triglyceride level peaked at 2050 mg/dL, and lipase was 52 U/L. The β-hydroxybutyrate level was obtained and found to be elevated at 5.29 mmol/L - the original sample was centrifuged and the chylomicron layer removed prior to analysis due to interference from turbidity caused by lipemia again. The patient was treated with an insulin drip for euDKA and HTG with a reduction in the anion gap to 13 and triglycerides to 1400 mg/dL, within 24 hours. Her euDKA was thought to be precipitated by her respiratory tract infection in the setting of SGLT2 inhibitor use.

The patient was seen by the endocrinology service and she was discharged on 40 units of insulin glargine at night, 12 units of insulin lispro with meals, and metformin 1000 mg two times a day. It was determined that all SGLT2 inhibitors should be discontinued indefinitely. She had close follow-up with endocrinology post discharge.

Limitations of this case

Many of the reported cases of euDKA in the literature have developed in patients with type-one diabetes mellitus, due to the off-label use of SGLT2 inhibitors, or in patients previously misdiagnosed as having T2DM, who, in fact, had the latent autoimmune diabetes of adults [1-7]. The limitations in the workup of our patient are that c-peptide, islet cell antibodies, or glutamic acid decarboxylase antibodies were not checked at any point during her diagnosis of diabetes mellitus.

## Discussion

We performed a literature search of PubMed using a combination of the words “euglycemic diabetic ketoacidosis,” “euglycemic DKA” with “SGLT2 inhibitors,” and “hypertriglyceridemia.” To our knowledge, this is the first case report in the literature, documenting DKA with normal glucose levels, in the setting of hypertriglyceridemia and SGLT2 inhibitor use. It is likely that the hypertriglyceridemia in our patient was incidental and contributed towards psuedonormoglycemia, as opposed to being an inciting factor for euglycemic DKA. The concomitant occurrence of DKA and hypertriglyceridemia is well-documented but there are only two case reports - one adult and one pediatric - of euDKA caused by hypertriglyceridemia alone [8-9]. Extreme hyperlipidemia can result in lipemic serum with a spurious lowering of blood glucose (pseudonormoglycemia) and serum sodium (pseudohyponatremia). The plasma concentration of glucose is decreased because of volume displacement by high levels of the circulating lipids. This may lead to a delay in the diagnosis of DKA [8-9]. SGLT2 inhibitors can increase low-density lipoproteins (LDL) cholesterol levels by decreasing its catabolism, however, there is no literature to suggest that SGLT2 inhibitors cause hypertriglyceridemia and, hence, we will regard these as independent factors in our patient’s euDKA [10]. We hypothesize that our patient’s normal blood glucose was due to a combination of hypertriglyceridemia and SGLT2 inhibitors since, in most case reports of isolated SGLT2-inhibitor-induced euDKA, blood glucose tends to be higher than normal but lower than 250 mg/dL [11].

SGLT2 inhibitors were approved in 2013 by the Food and Drug Administration (FDA) for the treatment of T2DM. They block the SGLT2 protein, which is involved in glucose re-absorption from the proximal renal tubule. This causes an increase in renal glucose excretion and a decrease in blood glucose levels [12]. There is strong evidence to suggest that SGLT2 inhibitors have beneficial effects on decreasing mortality from cardiovascular events, including a lower incidence of myocardial infarction and strokes [13-15]. SGLT2 inhibitors also cause weight loss without any risk of hypoglycemia [16]. In light of these desirable outcomes, SGLT2 inhibitors are an attractive class of medication for the management of hyperglycemia in T2DM.

However, there is a growing body of literature about the incidence of euDKA with SGLT2 inhibitors [1-7]. In 2015, the FDA released drug safety warnings about the risk of euDKA with the use of SGLT2 inhibitors [2,17].

In an observational study of clinical practices using a large insurance claims database, the unadjusted rate of diabetic ketoacidosis, within 180 days after the initiation of an SGLT2 inhibitor, was about twice the rate after the initiation of a dipeptidyl peptidase-4 inhibitor (4.9 vs 2.3 events per 1000 person-years) [6]. This has led to position statements by the American Associations of Clinical Endocrinologists (AACE) and the European Medicine Agency about the pathophysiology, prevention, diagnosis, and management of this unique disease entity [11,18].

The pathophysiology of euDKA with SGLT2 inhibitors is thought to involve the lowering of insulin production and increase the glucagon secretion, which promotes a shift of glucose to fat metabolism and stimulates ketogenesis [5,19-20]. SGLT2 inhibitors lower blood glucose levels by increasing urinary glucose excretion, which, in turn, reduces insulin secretion from pancreatic β‐cells. The decline in circulating insulin levels results in a lowering of the anti-lipolytic activity of insulin and consequent stimulation of the production of free fatty acids, which are converted to ketone bodies by β‐oxidation in the liver. Evidence suggests that the use of SGLT2 inhibitors stimulates the secretion of glucagon, either by a secondary effect mediated by the decrease in insulin secretion or by a direct effect on pancreatic α‐cells [19-20]. Another mechanism of euDKA is by the renal effects of SGLT2 inhibitors; during starvation, renal re-absorption of ketones increases with increase in serum ketone levels, with no apparent excretion threshold, but renal utilization of ketone bodies is reduced [19-20]. By lowering the renal glucose excretion threshold, SGLT-2 inhibition may mimic starvation conditions and cause an increase in ketone production and renal re-absorption [19]. Hence, SGLT2 inhibitors render the body susceptible to acidemia by ketogenesis and continue to produce glycosuria, thereby causing near normal or less abnormally elevated glucose levels than usually seen in DKA [11].

Per the 2016 American Association of Clinical Endocrinologists (AACE) position statement, patients with any form of diabetes who have abdominal pain, nausea, vomiting, fatigue, and/or dyspnea should be evaluated for DKA. Because SGLT2 inhibitors lower the threshold for glucose excretion, normal or modestly elevated blood glucose does not exclude the diagnosis of DKA during SGLT2-inhibitor use. Urine ketone monitoring is not ideal and is not recommended because it only measures acetoacetate, whereas the predominant ketone body in DKA is β-hydroxybutyrate. Therefore, the diagnosis should be confirmed with the direct measurement of the β-hydroxybutyrate level in blood and arterial blood pH [9]. Once the diagnosis is suspected, the SGLT2 inhibitor should be stopped immediately and DKA protocol initiated, including fluids, insulin, and other standard interventions [11].

EuDKA is typically precipitated by a reduction or omission of insulin, severe acute illness, dehydration, extreme physical activity, (e.g., running a marathon), surgery, low carbohydrate diets, or excessive alcohol intake [3,11]. Since the half-life of the different SGLT2-inhibitors is similar, ranging from 11 to 17 hours, SGLT2-inhibitor-associated euDKA can be prevented by the discontinuation of these medications at least three days before major surgical procedures, avoiding insulin omission or inappropriate insulin dose reduction and by following sick day protocols [3,11].

## Conclusions

In conclusion, euDKA caused by SGLT2 inhibitors is rare and largely caused in the setting of known precipitants. Hence, with adequate preventive measures, the risk associated with this disease entity can be minimized. Furthermore, since traditional markers of DKA can be absent in the setting of SGLT2 inhibitor use, all patients with signs and symptoms of DKA should be hospitalized and a full metabolic workup, including arterial pH and β-hydroxybutyrate level, must be checked to ensure that this diagnosis isn’t missed.

## References

[1] Monami M, Nreu B, Zannoni S, Lualdi C, Mannucci E (2017). Effects of SGLT-2 inhibitors on diabetic ketoacidosis: a meta-analysis of randomised controlled trials. Diabetes Res Clin Pract.

[2] GP Fadini, BM Bonora, A Avogaro (2017). SGLT2 inhibitors and diabetic ketoacidosis: data from the FDA Adverse Event Reporting System. Diabetologia.

[3] Goldenberg RM, Berard LD, Cheng AYY, Gilbert JD, Verma S, Woo VC, Yale JF (2016). SGLT2 inhibitor-associated diabetic ketoacidosis: clinical review and recommendations for prevention and diagnosis. Clin Ther.

[4] KR Burke, CA Schumacher, SE Harpe (2017). SGLT2 inhibitors: a systematic review of diabetic ketoacidosis and related risk factors in the primary literature. Pharmacotherapy.

[5] Ogawa W, Sakaguchi K (2016). Euglycemic diabetic ketoacidosis induced by SGLT2 inhibitors: possible mechanism and contributing factors. J Diabetes Investig.

[6] Fralick M, Schneeweiss S, Patorno E (2017). Risk of diabetic ketoacidosis after initiation of an SGLT2 inhibitor. N Engl J Med.

[7] Kim YG, Jeon JY, Han SJ, Kim DJ, Lee K, Kim HJ (2018). Sodium‐glucose co‐transporter 2 inhibitors and the risk for diabetic ketoacidosis in patients with type 2 diabetes mellitus: a nationwide population‐based cohort study. Diabetes Obes Metab.

[8] Akbay S, Yel A, Yıldırımer Ü, Can Ş, Dündar B (2013). Diabetic ketoacidosis presenting with pseudonormoglycemia in a 15-year-old girl with type 1 diabetes mellitus. J Clin Res Pediatr Endocrinol.

[9] Rumbak MJ, Hughes TA, Kitabchi AE (1991). Pseudonormoglycemia in diabetic ketoacidosis with elevated triglycerides. Am J Emerg Med.

[10] Briand F, Mayoux E, Brousseau E (2016). Empagliflozin, via switching metabolism toward lipid utilization, moderately increases LDL cholesterol levels through reduced LDL catabolism. Diabetes.

[11] Handelsman Y, Henry RR, Bloomgarden ZT (2016). Association of Clinical Endocrinologists and American College of Endocrinology position statement on the association of SGLT-2 inhibitors and diabetic ketoacidosis. Endocr Pract.

[12] Singh M, Kumar A (2018). Risks associated with SGLT2 inhibitors: an overview. Curr Drug Saf.

[13] Zinman B, Wanner C, Lachin JM (2015). Empagliflozin, cardiovascular outcomes, and mortality in type 2 diabetes. N Engl J Med.

[14] Mahaffey KW, Neal B, Perkovic V (2018). Canagliflozin for primary and secondary prevention of cardiovascular events: results from the CANVAS program (Canagliflozin Cardiovascular Assessment Study). Circulation.

[15] (2017). FDA approves Jardiance to reduce cardiovascular death in adults with type 2 diabetes. https://www.fda.gov/newsevents/newsroom/pressannouncements/ucm531517.htm.

[16] Clar C, Gill JA, Court R, Waugh N (2019). Systematic review of SGLT2 receptor inhibitors in dual or triple therapy in type 2 diabetes. BMJ Open.

[17] Food Food, U U (2019). Drug Administration Drug Safety Communication. FDA warns that SGLT2 inhibitors for diabetes may result in a serious condition of too much acid in the blood. https://www.fda.gov/drugs/drugsafety/ucm475463.htm.

[18] (2016). EMA confirms recommendations to minimise ketoacidosis risk with SGLT2 inhibitors for diabetes. https://www.ema.europa.eu/en/news/ema-confirms-recommendations-minimise-ketoacidosis-risk-sglt2-inhibitors-diabetes.

[19] Qiu H, Novikov A, Vallon V (2017). Ketosis and diabetic ketoacidosis in response to SGLT2 inhibitors: basic mechanisms and therapeutic perspectives. Diabetes Metab Res Rev.

[20] Bonner C, Kerr-Conte J, Gmyr V (2015). Inhibition of the glucose transporter SGLT2 with dapagliflozin in pancreatic alpha cells triggers glucagon secretion. Nat Med.

